# The Envelope Cytoplasmic Tail of HIV-1 Subtype C Contributes to Poor Replication Capacity through Low Viral Infectivity and Cell-to-Cell Transmission

**DOI:** 10.1371/journal.pone.0161596

**Published:** 2016-09-06

**Authors:** Eveline Santos da Silva, Martin Mulinge, Morgane Lemaire, Cécile Masquelier, Cyprien Beraud, Arkadiusz Rybicki, Jean-Yves Servais, Gilles Iserentant, Jean-Claude Schmit, Carole Seguin-Devaux, Danielle Perez Bercoff

**Affiliations:** 1 Department of Infection and Immunity, Luxembourg Institute of Health, 29 rue Henri Koch, L-4354 Esch-sur-Alzette, Luxembourg; 2 Centre Hospitalier de Luxembourg, Service National des Maladies Infectieuses, 4 Rue Ernest Barblé L-1210 Luxembourg, Luxembourg; Institut Pasteur, FRANCE

## Abstract

The cytoplasmic tail (gp41CT) of the HIV-1 envelope (Env) mediates Env incorporation into virions and regulates Env intracellular trafficking. Little is known about the functional impact of variability in this domain. To address this issue, we compared the replication of recombinant virus pairs carrying the full Env (Env viruses) or the Env ectodomain fused to the gp41CT of NL4.3 (EnvEC viruses) (12 subtype C and 10 subtype B pairs) in primary CD4+ T-cells and monocyte-derived-macrophages (MDMs). In CD4+ T-cells, replication was as follows: B-EnvEC = B-Env>C-EnvEC>C-Env, indicating that the gp41CT of subtype C contributes to the low replicative capacity of this subtype. In MDMs, in contrast, replication capacity was comparable for all viruses regardless of subtype and of gp41CT. In CD4+ T-cells, viral entry, viral release and viral gene expression were similar. However, infectivity of free virions and cell-to-cell transmission of C-Env viruses released by CD4+ T-cells was lower, suggestive of lower Env incorporation into virions. Subtype C matrix only minimally rescued viral replication and failed to restore infectivity of free viruses and cell-to-cell transmission. Taken together, these results show that polymorphisms in the gp41CT contribute to viral replication capacity and suggest that the number of Env spikes per virion may vary across subtypes. These findings should be taken into consideration in the design of vaccines.

## Introduction

Spread of Human Immunodeficiency Virus (HIV-1) to new target cells *in vitro* and *in vivo* occurs via infection with free virions or by direct transmission of newly synthesized virions budding from an infected “donor” cell to a nearby target cell [1–8] reviewed in [9, 10]. Both modes of infection are mediated by the viral envelope (Env). Env is a highly glycosylated trimeric complex composed of a surface subunit (gp120) and a transmembrane anchoring subunit (gp41) which are non-covalently linked [11]. The 2 Env subunits result from the proteolytic cleavage of the trimeric gp160 precursor protein by the cellular protease Furin in the Golgi apparatus [12, 13]. The surface subunit gp120 ensures viral adsorption and binding to the CD4 receptor [14–16] and the coreceptor (CCR5 or CXCR4) [17–20]. These interactions induce a series of conformational changes in Env and lead to the insertion of the fusion peptide located at the N-terminus of the transmembrane subunit gp41 into the target cell membrane and to fusion of the viral and cellular membranes [21–27]. The Env surface subunit gp120 and the extracellular portion of gp41 have been extensively studied, but the cytoplasmic domain of Env (gp41CT) has been granted far less attention and many of its functions remain poorly understood or speculative.

The gp41CT of lentiviruses, including HIV-1, is unusually long (~150 amino acids (AA)) in comparison to other retroviruses (< 50 AA) [28]. Immediately downstream of the membrane-spanning domain (MSD) lies the immunodominant Kennedy polypeptide sequence followed by three highly conserved α-helix domains referred to as the lentivirus lytic peptides (LLP): LLP-2 (AA 773–793) which overlaps the LLP-3 leucine zipper domain (AA 785–807), and LLP-1 (AA 833–856) [29]. Despite considerable sequence variation, the physicochemical and structural properties of peptides spanning the LLP regions are believed to be conserved across HIV types and HIV-1 subtypes [30].

The gp41CT of lentiviruses ensures several functions (recently reviewed in [31–35]). The main function of the gp41CT is to ensure Env packaging into nascent virions, by interacting with the matrix protein (MA) at the N-terminus of the p55Gag precursor [36–48]. The MA/gp41CT interaction maps to charged AA within LLP-2 and to the P_T/R_RIR domain of LLP-1 [38–44], but whether it is direct or indirect remains a matter of debate [31, 49–56]. The gp41CT also regulates Env trafficking to and from the plasma membrane (PM) through the Trans Golgi Network (TGN). As soon as it reaches the membrane, Env is internalized [57–59], following its interaction with the AP-2 μ (medium) chains via the highly conserved Y_712_SPL [60–62] and the C-terminal LL_856_ [63, 64] Golgi retrieval signals. Endocytosed Env can either proceed to be degraded by lysosomes or be sorted back to the Golgi by interacting with retromer components Vps26 and Vps35 via is1 and is2 [65] or with a number of other proteins which regulate its traffic through the TGN and back to the PM. These include TIP47 through the Y_802_W_803_ diaromatic motif [50–54], AP-1 and AP-3 through the Y_712_SPL and the C-terminal dileucine LL_856_ motifs [61, 63, 64] and Rab11a/FIP1C and Rab14 through the YW_795_ diaromatic motif [55, 66]. AP-2-mediated internalization of Env is reversed by the p55Gag polyprotein precursor, and it was proposed that Env internalization is a means to evade immune recognition and proceeds until sufficient Gag has assembled at the PM to trap Env into the budding virion [67, 68]. The gp41CT also contributes to viral infectivity of Env and to cell-to-cell transmission by maintaining the structure of Env [69–73]. The gp41CT Y_712_SPL motif induces polarization of p55Gag at the basolateral membrane in polarized non-human cell lines [74–76] and in T-cells [73, 77]. In the mature virion, it enables Env clustering, a prerequisite of efficient infectivity [78–82]. Last but not least, the gp41CT was reported to favor viral transcription through direct and indirect mechanism. Recently, the gp41CT of HIV and of SIV have been shown to contribute to cell activation by inducing the canonical NF-κB pathway through Y_768_ within the YHRL motif of LLP-2 [83]. Indirect mechanisms involve the relief of the RhoA-mediated transcriptional inhibition by interacting with p155-RhoGEF [84, 85] and induction of LTR-driven transcription by targeting the precursor of luman, a repressor of Tat-mediated HIV transcription, for proteasomal degradation [86].

gp41CT-truncated viruses display a cell-type dependent phenotype and are unable to replicate in a number of T cell lines (H9, MT-2, Jurkat and CEMx174) and in primary target cells (primary CD4+ T-cells and macrophages), referred to as “non-permissive cells”. In other cell lines (MT-4 and M8166, HEK, HeLa, and COS), in contrast, the impact of such truncations is limited (“permissive cells”) [38, 39, 42, 44, 73, 87–89]. The non-permissive phenotype has been associated with decreased Env incorporation into virions [42–44, 70, 90, 91], irrespective of the levels of Env at the cellular PM [41, 42, 87, 88].

Env variability and subtype-related specificities have long been acknowledged in studies investigating the Env ectodomain and in vaccine design. Yet most mutational or biochemical studies addressing the role of the gp41CT have been performed using the laboratory-adapted strain NL4.3 with mutations in charged residues or disrupted dileucine motifs. Few studies have addressed the role of polymorphisms in the gp41CT. One biochemical study suggests its biophysical properties are likely conserved despite type and subtype specific polymorphisms [30] and two studies have investigated the impact of specific mutations in the Y_712_SPL and Y_802_W_803_ trafficking motifs [53] or of LLP-1 truncations occurring in patient samples on viral replication and Env intracellular distribution, as well as the role of MA in rescuing such defects [91]. Studies examining the functional impact of non-subtype B polymorphisms on viral replication and virion infectivity are missing. Here we compared the infectivity of viruses with subtype C Envs to viruses with the extensively studied subtype B envs in primary CD4+ T-cells and monocyte-derived-macrophages (MDMs) *in vitro*. Subtype C is the most widely spread subtype, accounting for > 52% of infections worldwide [92]. In vitro, subtype C viruses have lower replication capacity than subtype B or D viruses, owing to Env [93–97]. We therefore enquired whether the gp41CT also contributed to the lower replication capacity of subtype C viruses *in vitro*. Using recombinant virus pairs differing only by their gp41CT in an otherwise constant viral backbone, we found that CD4+ T-cells but not MDMs infected with viruses harboring subtype C Envs produced less virions than viruses with subtype B Envs. The gp41CT substantially accounted for this defect in propagative infection as it enabled less Env incorporation into virions and poor cell-to-cell transmission. Subtype C MA only minimally restored replication, and failed to rescue virion infectivity and cell-to-cell transmission, suggesting subtype-related differences.

## Materials and Methods

### Cell lines

HEK 293T and TZM-bl cells (ATCC through the NIH AIDS Research and Reference Reagent Program) were maintained in Dulbecco’s Modified Eagle Medium (DMEM) supplemented with 10% Fetal Calf Serum (FCS), 2 mM L-Glutamine, 50 μg/mL Penicillin and 50 μg/mL Streptomycin (all from Invitrogen, Merelbeke, Belgium).

### Isolation and culture of primary cells

Human peripheral blood mononuclear cells (PBMCs) were isolated from buffy coats obtained from healthy volunteer HIV-1 seronegative donors (Red Cross Luxembourg) by Lymphoprep density gradient centrifugation (Axis-Shield, Oslo, Norway). CD4+ T-cells were isolated from PBMCs by negative selection using antibody coated-beads (Miltenyi Biotec, Leiden, Netherlands) and cultured in RPMI 1640 medium supplemented with 10% FCS, 2mM L-Glutamine, 50 μg/mL Penicillin and 50 μg/mL Streptomycin (all from Invitrogen, Merelbeke, Belgium) (CD4 medium). PBMCs were stimulated with 5 μg/ml PHA-P (Sigma, Borneim, Belgium) for 48 hours, then with 10 U/ml interleukin 2 (IL-2, Roche, Mannheim, Germany) for 24 hours before infection.

Monocytes were isolated from fresh PBMCs by adherence to plastic for 1 hour at 37°C in MDM medium (RPMI 1640, 2mM L-Glutamine, 50 μg/mL Penicillin and 50 μg/mL Streptomycin, 10 mM HEPES, 1% MEM vitamins, non-essential amino acids, 50 μM β-mercaptoethanol, 1 mM sodium pyruvate) (all from Invitrogen, Merelbeke, Belgium) supplemented with 2% human AB serum (Sigma, Bornem, Belgium) as described in [98]. Adherent monocytes were seeded in 96-well plates (3x10^5^ cells/well) and allowed to differentiate into macrophages for 7 days in MDM medium supplemented with 15% human AB serum.

### Viral Samples

Plasma samples from 22 treatment-naive patients infected with HIV-1 subtypes B (10 patients) and subtype C (12 patients) were included in the study. Ethical approval was obtained from the Comité National d’Ethique pour la Recherche in Luxembourg (approval number: 201105/07). Patients provided written informed consent. Mean plasma viral load (VL) (Abbott m2000 RealTime HIV-1 assay) and CD4 counts were comparable for patients infected with either subtype: mean VL for subtype B was 34181 RNA copies/ml [range: 635–124657] and mean VL for subtype C was 38223 RNA copies/ml [range: 1759->500000]; mean CD4 count was 386 cell/mm^3^ [range: 110–610] for subtype B-infected patients and 368 [range: 100–760] for subtype C-infected patients. HIV-1 subtype was assigned from HIV-1 PR-RT and Env sequences (V3-loop and gp41CT) using COMET [99] and the REGA HIV subtyping tool [100]. A phylogenetic tree (GTR+G model) with 1000 bootstrap values was inferred for the PR-RT and Env gp41CT sequences with RAxML version 7.6.0 using reference strain G.BE.96.DRCBL.AF084936 as the outgroup. Phylogenetic analysis confirmed the samples were unrelated (data not shown). Coreceptor usage was determined as described in [101]. Subtype B samples included 7 strictly R5, 1 R5/X4 and 2 strictly X4 strains; 8 subtype C strains were strictly R5 and 4 were R5/X4. Two reference clones with identical gp41CTs, NL4.3 (X4 tropic) [102] and NLAD8 (R5 tropic) [103], were included as controls in all experiments. Of note, NLAD8 contains the ectodomain of ADA and the gp41CT from NL4.3, downstream of the BamHI site [103].

### Env amplification

One ml of HIV-1 positive plasma was centrifuged at 24 000g for 1 hour at 4°C and viral RNA was extracted from the pellet using the Qiagen Viral RNA extraction kit (Qiagen, Hilden, Germany). Viral cDNA was synthesized in a one-step RT-PCR reaction using forward primer KVL008 and reverse primer KVL009 [104] as described in [101]. A nested PCR was performed from 2 μl of cDNA using forward primer rec.envHXB2_Fp (5′-TAGGCATYTCCTATGGCAGGAA'-3’) and reverse primer rec.HR1-2_RP (5’-CTCTCTCTCCACCTTCTTCTTC-3’) to amplify the Env ectodomain (EnvEC, gp140) or subtype-dependent reverse primers Rp-fullEnv-B (5’-TCGTCTCATTCTTTCCCTTAC-3’) and Rp-fullEnv-C (5’-TCMTCTYATTCTTTCYCTTAC-3’) respectively for subtype B and C the full Env sequence (Env, gp160). Nested PCR reactions were performed using 2.5 U Platinum *Taq* High Fidelity over 35 cycles (95°C for 30 sec, 48°C for 30 sec and 68°C for 3 min), and a final 10 min extension at 68°C. Amplification was verified by agarose gel electrophoresis. To avoid PCR selection, for each sample five independent amplifications were performed in parallel and pooled. Gp41CT sequences are available under GenBank accession numbers HG313608-HG313634.

### Construction of viral backbones

**Fig 1:**
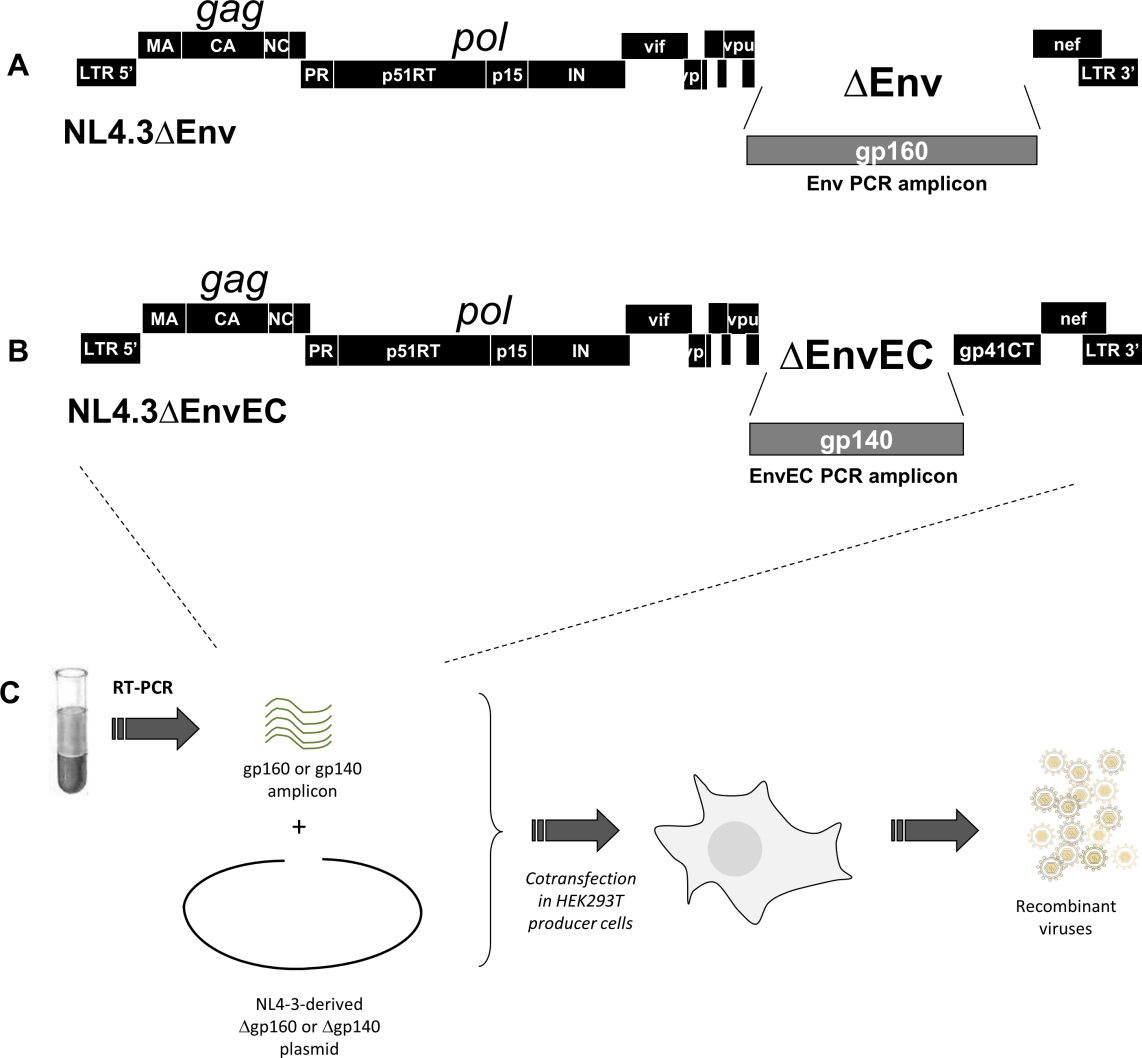
Generation of recombinant viruses and molecular constructs pNL4.3ΔEnv and pNL4.3ΔEC. **A and B:** The molecular constructs were based on the pNL4.3 infectious clone deleted of the entire Env (gp160, ΔEnv) **(A)** or of the Env gp120+gp41 ectodomain (gp140, ΔEnvEC) **(B)** by inverse PCR. The deleted portion is replaced by the *AfeI* restriction site for linearization prior to transfection. **C**: Infectious viral particles expressing patient-derived Env or EnvEC sequences were generated by co-transfecting HEK 293T cells with the linearized pNL4.3ΔEnv or pNL4.3ΔEC backbones and a PCR amplicon spanning the target region (gp160 (Env) or gp140 (EnvEC) respectively).

pNL4.3ΔEnv (Fig 1A) and pNL4.3ΔEnvEC (Fig 1B) were generated by deleting the full *env* or the *env ectodomain* (EnvEC) from pNL4.3 respectively by inverse PCR and replacing it by a *AfeI* restriction site for linearization. To construct pNL4.3ΔEnv, the *EcoRI-XhoI* fragment from pNL4.3 was subcloned into pBluescript KS+ (Stratagene, Amsterdam, Netherlands), the entire *envelope* (gp160) was deleted from pBluescript.Env by inverse PCR using phosphorylated primers Rp_del.full.Env: 5'-GCTGTCTTCTGCTCTTTCTATTAG-3' and Fp_del.full.Env: 5'-GCTATGGGTGGCAAGTGGTC-3' designed to introduce a *AfeI* restriction site (underlined), *DpnI* digested and ligated to create pBluescriptΔEnv.AfeI. The *EcoRI–XhoI* fragment from pBluescriptΔEnv.AfeI was transferred back to pNL4.3. To construct pNL4.3ΔEnvEC, the *SalI-XhoI* fragment from pNL4.3ΔEnv.EC.Luc [105] was cloned into pNL4.3. All constructs were sequenced.

To introduce subtype C MA into pNL4.3ΔEnv, the *matrix* sequences of subtype C strains 1671 and 0266 were amplified using forward primer 0266-BssHI-F: 5’-ACTCGGCTTGCTGAAGCGCGCTCG-3’ containing *BssHII* (underlined) and reverse primer 0266-SphI-R: 5’-TGCATGCAGGGCCTRTTGCACC-3’ containing the *Sph*I restriction site (underlined). The BssHII-SphI digested PCR product was cloned into the BssHII-SphI digested pNL4.3ΔEnv backbone and the constructs were sequenced after cloning to ensure conformity of the cloned MA to the bulk sequence.

### Production of Env and EnvEC Recombinant Viruses

To produce recombinant viral particles, 2.5x10^6^ HEK 293T cells were cotransfected with 8 μg of *AfeI-*linearized pNL4.3ΔEnv or pNL4.3ΔEnvEC backbones and patient-derived Env PCR amplicons (10 μl of EnvEC amplicon and 30 μl full Env amplicon) (Fig 1C) using Lipofectamine 2000 (Invitrogen, Merelbeke, Belgium). Cell-free culture supernatants were collected 48 hours later, clarified by centrifugation and stored at -80°C until use. Viral production was determined by quantifying p24 capsid protein using a p24 ELISA test (Innogenetics, Belgium). Amplicons produced from pNL4.3 (X4) and pNLAD8 (R5) plasmids were used as positive controls and viral-like particles lacking an envelope produced by transfecting the linearized backbones alone were used to assess background noise.

### Infections

Activated CD4+ T-cells (10^5^ cells/well) or MDMs (3x10^5^ cells/well) were infected in 96-well plates with normalized amounts of Env and EnvEC recombinant virus pairs (1 ng/ml (CD4+ T-cells) or 3 ng/ml (MDM) equivalent p24) by spinoculation (1200 g for 2 hours at 25°C), followed by a 1 hour- (for CD4+ T-cells) or 2 hour- (for MDMs) incubation at 37°C and washing. Viral supernatants were collected every 3–4 days and stored at -80°C until use. All infections were performed in triplicate or quadruplicate wells. Viral replication was assayed by measuring p24 in culture supernatants by ELISA (Innogenetics, Belgium). Because gp41CT truncations in primary CD4+ T-cells and in non-permissive T-cells lines can be overridden by high viral gene expression such as those reached at saturating viral inputs [73], preliminary experiments with 10 ng/ml, 3 ng and 1 ng/ml were conducted to identify viral inputs that reproducibly infected primary CD4+ T-cells and MDMs without saturating the system (data not shown). For some control experiments, 10 μM AZT (Sigma, Bornem, Belgium) were added to ensure only *de novo* viral replication was measured. In some experiments, polybrene (5 μg/ml) was added at the time of infection.

TZM-bl cells (HeLa-derived cells expressing CD4, CCR5 and LTR-luc) (2x10^4^ cells/well) were infected with 2 ng/ml equivalent p24 in the same conditions as CD4+ T-cells or MDMs and luciferase was measured in cell lysates 48 hours post-infection using the Promega luminescence kit.

### Virus tethered to the PM

To measure virus tethered to the PM, CD4+ T-cells were infected for 5 days as above, washed once with PBS and incubated for 15 min at 37°C in 100 μl of Tris/HCl (pH 8.0) with 150 mM CaCl_2_ with or without 1 mg/ml subtilisin (Sigma). The reaction was stopped with PMSF (5mM in 0.5 ml FCS-supplemented culture medium) and p24 in each fraction was measured by ELISA. The amount of virus released after protease treatment was related to total virus, i.e. p24 released in viral supernatants+p24 bound to the PM.

### Cell-to-cell transmission

CD4+ T-cells (4x10^5^/well) in sextuplicates were infected with 1 ng/ml recombinant C-Env or C-EnvEC viruses by spinoculation as described above. After 48 hours, CD4+ T-cells (donor cells) were washed extensively and co-cultured with TZM-bl target cells for 48 hours. 1 μM indinavir (IDV) was added in half the wells to control for infection by free virus. The next morning, 1 μM AMD3100 and 1μM TAK-779 were added to limit replication to a single cycle. Transmission to TZM-bl cells was assessed by measuring luciferase after removal of CD4+ T-cells and washing of target cells. To control for the contribution of free virus, the supernatant of the co-cultures was collected and used to infect TZM-bl cells.

### Western Blot

To monitor Env incorporation into viral particles, supernatants from transfected HEK 293T cells and from infected CD4+ T-cells were purified on a sucrose cushion composed of a 10%-60% sucrose cushion using a Beckman Optima MAX Ultracentrifuge at 50 000 rpm for 16 hours at 4°C. The fractions containing purified virions were harvested and concentrated at 25 000 rpm for 16 hours at 4°C. Virus content in CD4+ T-cell supernatants was too low to be detected by ELISA, WB or silver staining despite repeated attempts. Ultracentrifuged crude supernatant was therefore used. The samples were lysed at 95°C for 10 min with reducing Laemmli buffer and resolved on 10% SDS-PAGE gels. For immunoblotting, proteins were transferred to nitrocellulose membranes and probed overnight at 4°C with goat anti-HIV-1 gp120 (ab53937) and mouse anti-HIV-1 p24 clone 39/6.14 (Abcam, Cambridge UK) antibodies. Secondary antibodies were donkey anti-goat and rabbit anti-mouse IgG conjugated to HRP (Sigma-Belgium).

For analysis of intracellular viral proteins, triplicate wells of infected CD4+ T-cells were washed and pooled. 10 μg of protein were mixed with reducing Laemmli buffer and resolved on 12% SDS-PAGE gels. For immunoblotting, rabbit α-p55Gag+p17+p24 ab63917 (1/1000) (Abcam), goat anti-HIV-1 gp120 (ab53937) and rabbit anti-β-actin (Cell Signaling #49675) were used. After washing, membranes were incubated sequentially with donkey anti-goat IgG, then with goat anti-rabbit IgG, both conjugated to HRP (Sigma-Belgium) and developed using ECL (Amersham).

Images were acquired with a GE Healthcare ImageQuant LAS 4000 and bands corresponding to viral proteins in each lane were quantified using ImageJ and normalized to β-actin to estimate their relative amount.

### Statistical analyses

Statistical analyses were performed using GraphPad Prism v 5.01, applying a paired t-test for pairs of EnvEC and Env viruses within a given subtype (i.e C-EnvEC and C-Env viruses or B-EnvEC and B-Env viruses) and a Kruskal-Wallis test for comparisons between subtypes (e.g. C-EnvEC and B-EnvEC viruses, C-Env and B-Env viruses, C-EnvEC and B-Env viruses). Statistical relevance was considered if p < 0.05. The standard deviation between results from independent experiments is reported.

## Results

### The gp41CT contributes to the lower replication of subtype C viruses in CD4+ T-cells but not in MDMs

**Fig 2:**
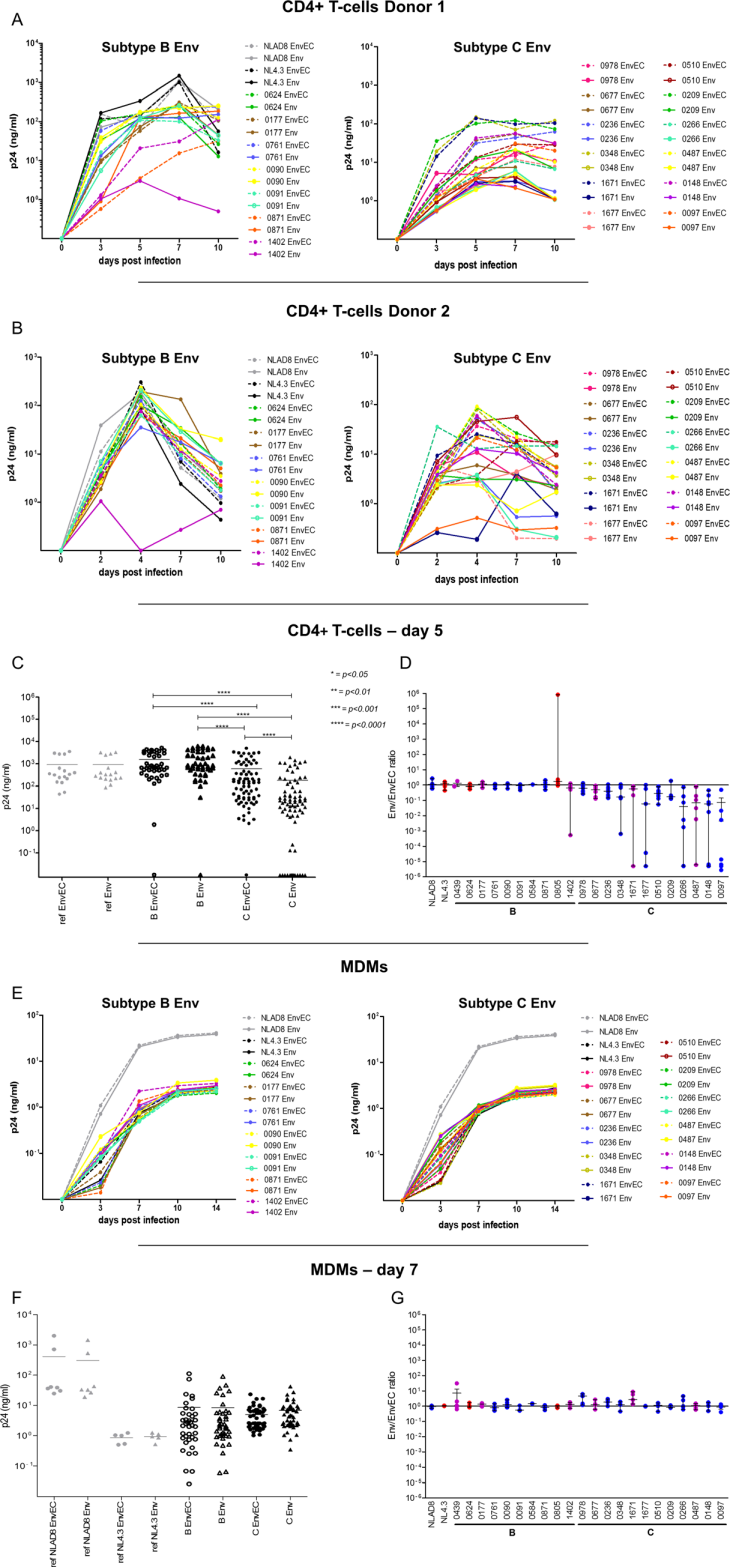
Viruses with subtype C gp41CT have lower replication than viruses with subtype B gp41CT in CD4+ T-cells but not in MDMs. **A and B. Replication of subtype B and subtype C recombinants in CD4+ T-cells from two different donors.** 10^5^ CD4+ T-cells were infected with equivalent amounts (1 ng/ml) of Env or EnvEC recombinant viruses. Infection was monitored by measuring p24 in culture supernatants at days 2 or 3, 4 or 5, 7 and 9 or 10. Examples of replication in CD4+ T cells of two different donors are shown after infection with subtype B (left panels) and subtype C (right panels) Env and EnvEC recombinant viruses. All infections were performed in triplicate wells. **(C and D) P24 in supernatants of 10**^**5**^ **CD4+ T-cells 5 days post-infection.** The same results at day 5 post-infection are presented and statistically significant means (paired t-test for viral pairs, unpaired t-test for inter-subtype comparisons) are indicated (**C**). In panel (**D**), the results are shown per pair. The ratio of p24 released in supernatants of cells exposed to Env recombinant viruses divided by p24 in the supernatants of cells exposed to the corresponding EnvEC recombinant virus is reported. A positive ratio indicates that the full Env recombinant replicates better than its EnvEC counterpart containing the gp41CT of NL4.3. A negative ratio implies the EnvEC recombinant virus replicates better than the Env recombinant. Because of inter-donor variability, results are presented as individual experiments performed with CD4+ T-cells from independent donors. **E. Replication of subtype B and subtype C recombinants in MDMs.** 10^5^ MDMs were infected with equivalent amounts (3 ng/ml) of subtype B (left panel) and subtype C (right panel) Env and EnvEC recombinant viruses. Infection was monitored by measuring p24 in culture supernatants at days 3, 7, 10 and 14. All infections were performed in triplicate or quadruplicate wells. **(F and G) p24 in supernatants of 3x10**^**5**^ **MDMs 7 days post-infection.** All infections were performed in triplicate or quadruplicate wells. Standard deviation is indicated. In panel (**G**), the same results as in panel (F) are represented for each individual strain. The ratio of p24 released in supernatants of cells exposed to Env recombinant viruses divided by p24 in the supernatants of cells exposed to the corresponding EnvEC recombinant virus is reported. Tropism is indicated as follows: R5: blue circle; X4: red circle; R5X4: purple circle.

To assess whether naturally occurring polymorphisms in the gp41CT impact viral replication, we used a comparative approach based on NL4.3-derived paired recombinant viruses containing patient-derived sequences spanning the full *env* (gp160 –Env viruses) or the *env ectodomain* and *transmembrane domain* (gp140 –EnvEC viruses) (Fig 1). With this approach, replicating viral particles differing only by the gp41CT (patient-derived vs NL4.3-derived) and reflecting the heterogeneity of quasispecies are obtained. Twelve subtype C and 10 subtype B patient-derived strains were included. To validate the system, we verified that Env and EnvEC virus pairs carrying the Env or EnvEC of the controls NL4.3 and NLAD8 had similar replication in CD4+ T-cells (Fig 2A–2D) and in MDMs (Fig 2E–2G), given that both carry the gp41CT from NL4.3.

Viral replication was followed over 10 days. As can be seen from Fig 2A and 2B, infection of CD4+ T-cells with subtype B viruses did not show great variability within pairs, regardless of the gp41CT, while for viruses with subtype C Envs, C-Env virus released in supernatants remained lower than the corresponding C-EnvEC virus. Replication of viruses with subtype C gp41CT plateaued after day 5 and the difference in viral replication was amplified over time, suggestive of hindered propagative infection. To ease comparison and to include infection experiments with sufficient different donors, viral production in CD4+ T-cell culture supernatants was compared 5 days post-infection. Production of viruses with subtype B Env or EnvEC at day 5 post-infection was comparable and was significantly higher than replication of C Env and C-EnvEC viruses in the following order B-Env = B-EnvEC > C-EnvEC > C-Env viruses at all time points (Fig 2C and 2D). For subtype C Env pairs, at this time-point viral production of C-Env viruses was 5-10-fold lower than their chimeric C-EnvEC counterpart in 6/12 cases (“intermediate” replication capacity), and in 5/12 cases it was over 10-fold lower (“low” replication capacity) (Fig 2D). In one case (strain 0978), replication was similar for both or higher for C-Env viruses, depending on donor cells (Fig 2D).

In MDMs, in contrast, no difference between subtypes (p>0.05, unpaired t-test) nor between paired recombinants was recorded (Fig 2E) over 14 days of culture (p>0.05, paired t-test) (Fig 2F and 2G), indicating that the subtype of the gp41CT did not modify replication levels in this cell type. AZT fully abrogated viral replication in MDMs (not shown), confirming that p24 detected in MDMs corresponded to *de novo* viral production and not to virus inoculum bound to cells.

These results confirm that the viral Env is an essential driver of viral replication [94, 95, 97, 106] and show that not only the Env ectodomain, but also the gp41CT contributes to viral replication capacity in CD4+ T cells *in vitro*. These results also show that in macrophages, replication is comparable regardless of Env and of the gp41CT. Sequence analysis (S1 Fig) showed conservation of subtype B gp41CT and highlighted subtype C-specific polymorphisms. Yet no obvious link between polymorphisms and replication levels could be identified.

### Lower viral replication of C-Env viruses is not due to lower viral gene expression

**Fig 3:**
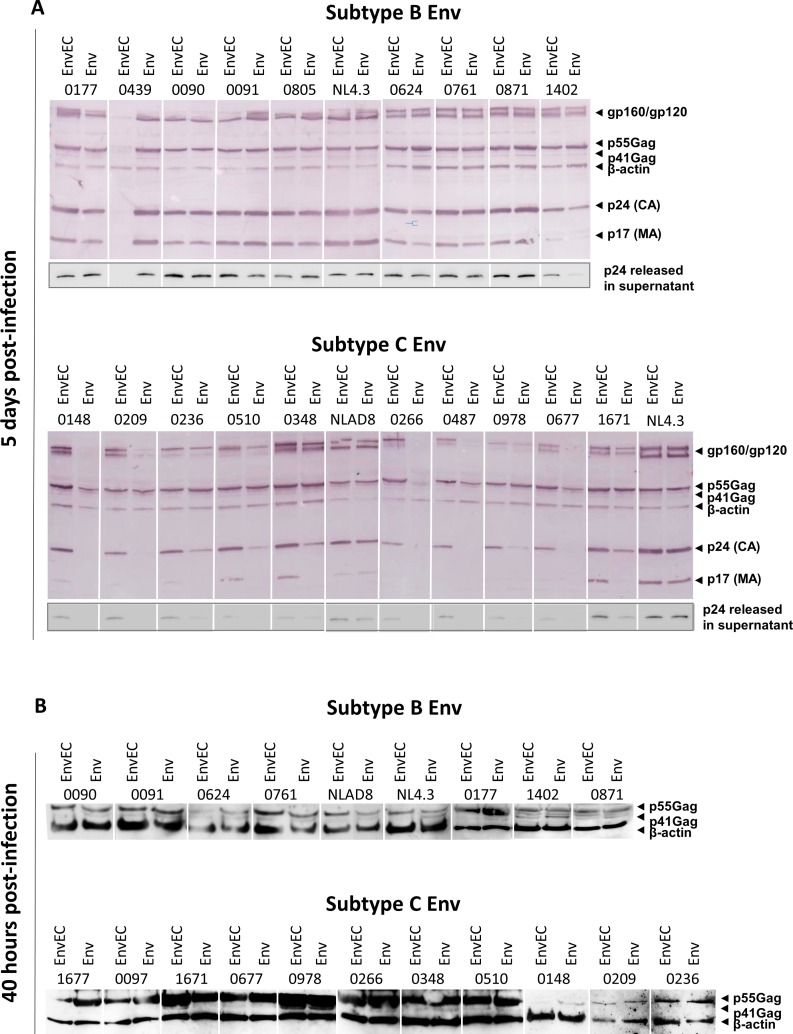
Lower replication of C-Env recombinants in CD4+ T-cells is not associated with lower viral gene expression. **A and B. Western Blot analyses of intracellular Env and Gag in CD4+ T-cells exposed to subtype B and subtype C recombinant virus pairs at day 5 post infection (A) and 40 hours post infection (B).** CD4+ T-cells (10^5^ cells/well) infected in triplicate wells for 5 days (A) or for 40 hours (B) were washed, pooled and lysed in 75μl of reducing Lämmli buffer. Polybrene was added at the time of infection of C-EnvEC and C-Env virions for WB detection in 40-hour lysates. Proteins were resolved by SDS-PAGE and immunoblotted with a rabbit polyclonal antibody recognizing the immature p55Gag immature precursor and the mature p17 and p24 proteins, and a goat polyclonal antibody against gp120. β-actin was immunoblotted to control for loading. The corresponding supernatant (30 μl) were immunoblotted using a mouse monoclonal antibody against p24. One representative experiment of three is shown. Samples are arranged by subtype to ease visual comparison. C and B Env samples were also run on the same gel, and similar subtype and gp41CT-related differences were observed (not shown).

To gain some insight into the lower viral replication of C-Env viruses in CD4+ T-cell cultures, viral protein levels and profiles were examined at day 5 post-infection. As can be seen from Fig 3A, cells infected with B-EnvEC and B-Env viruses displayed comparable viral protein levels and profiles, while cells infected with C-Env and C-EnvEC viruses showed lower viral protein levels. More specifically, whereas different Gag forms (p55Gag, p41Gag, p24 and p17) and Env (gp160 and gp120) were readily detected in CD4+ T-cells infected with subtype B viruses and C-EnvEC viruses, only p55Gag and p41Gag were detected in CD4+ T-cells infected with C-Env viruses, while mature forms of Gag (p24 and p17) and Env (gp160 and gp120) remained consistently lower or undetectable (Fig 3B). β-actin levels did not fluctuate with mature forms of Gag or with Env levels, arguing against higher cell death. Furthermore, cell death, as measured with an MTT assay, correlated with p24 measured in culture supernatants (not shown). Therefore, the lower viral protein levels recorded in day-5 cell lysates likely reflect lower or slower viral and are in line with the lower viral production recorded at this time point (Fig 2A–2D). Accordingly, p24 released in the supernatant paralleled intracellular mature forms of Gag (p24+p17) for all recombinant viruses at day 5 (Spearman r = 0.5683, p<0.0001) (Fig 3A).

It has been suggested that low intracellular mature forms of Gag reflect low gene expression from the LTR [73]. Moreover, the gp41CT was reported to de-repress viral transcription from the LTR [84–86] and to induce nuclear translocation of NF-κB p65/RelA [83]. To assess whether low viral protein expression underlies the lower viral production recorded in CD4+ T-cell cultures infected with C-Env viruses or whether lower viral protein expression merely reflect lower or slower viral replication, proteins in CD4+T-cell lysates were quantified 40 hours post infection. Polybrene had to be added to CD4+ T-cells exposed to C-EnvEC and C-Env viruses because in its absence, no p55Gag could be detected for these viruses at this early time point. As shown in Fig 3B, the expression of the viral gene product p55Gag was comparable between C-EnvEC and C-Env viruses.

In MDM lysates, viral replication levels were too low to be detected by WB (not shown).

Taken together, these observations argue against the possibility that differences in viral protein expression account for the differences in viral production we recorded and suggest that lower viral protein expression rather reflects a delay of a defect in later steps of the viral replication cycle.

### Viral release is not impaired

**Fig 4:**
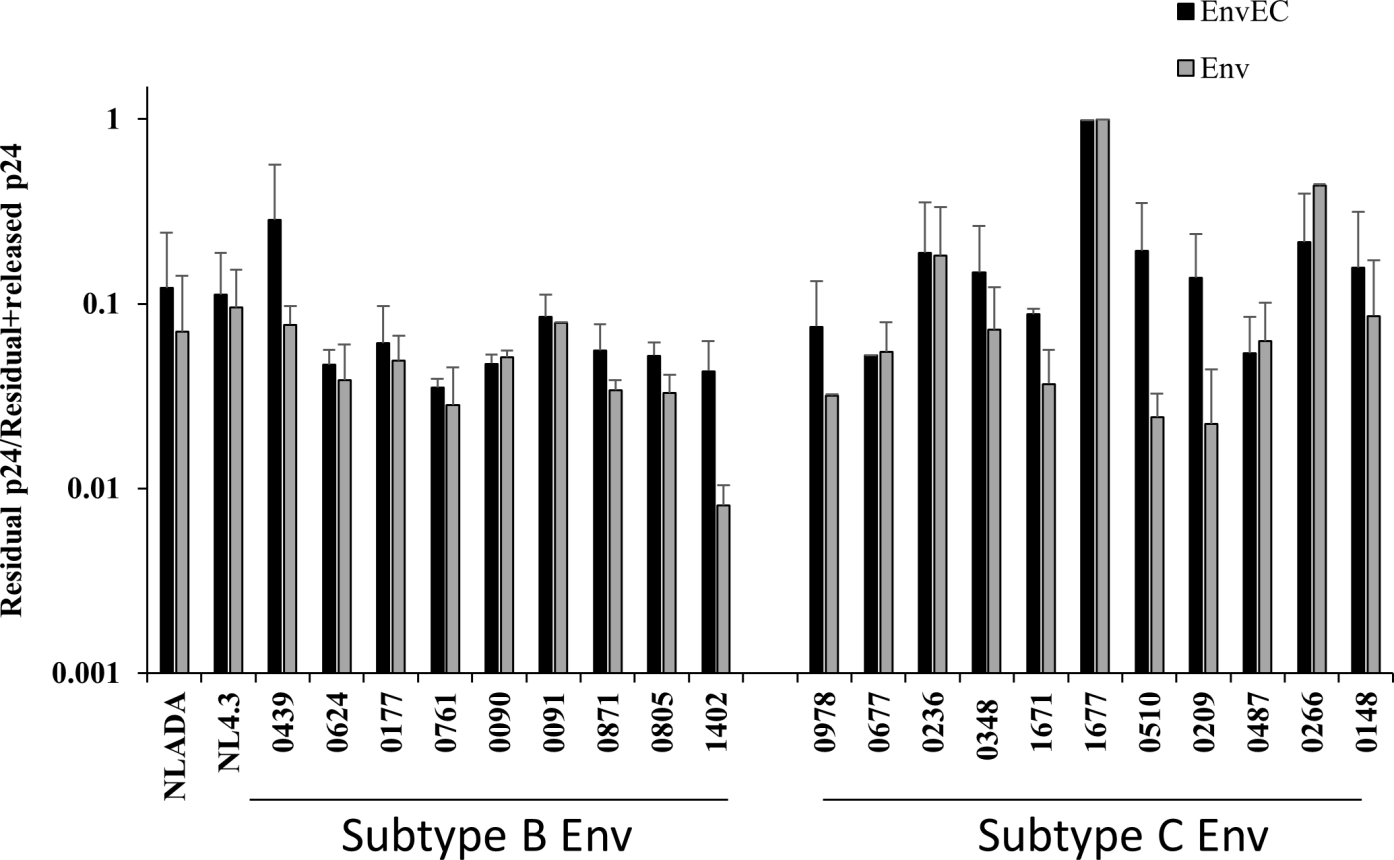
The gp41CT of subtype C Envs does not tether virus to CD4+ T-cells. CD4+ T-cells (10^5^ cells/well) were infected with Env or EnvEC recombinant virus pairs for 5 days, treated with subtilisin or with buffer alone, and virus in each fraction was measured by p24 ELISA. The amount of p24 that released upon subtilisin-treatment was reported to total p24 (i.e. released in the supernatant+virus attached to the PM). Three experiments with CD4+ T-cells from different donors are shown (average). Error bars represent standard deviation.

We next considered the possibility that viruses might remain tethered at the PM, consequent to intrinsic properties of virions with subtype C gp41CT or to recombinant B-C Vpu. To investigate this hypothesis, virus tethered to the PM of CD4+ T-cells was released by subtilisin digestion. Fig 4 illustrates that there was no difference in the amount of virus which remained attached to CD4+ T-cells infected with EnvEC or with the corresponding Env recombinant for subtype B or for subtype C Envs, arguing against the possibility that the observed phenotype is a Vpu-related bias or involves a tetherin-like activity of subtype C gp41CT interfering with viral release.

### C-Env virions produced by CD4+ T-cells, but not by HEK293T cells, are less infectious

**Fig 5:**
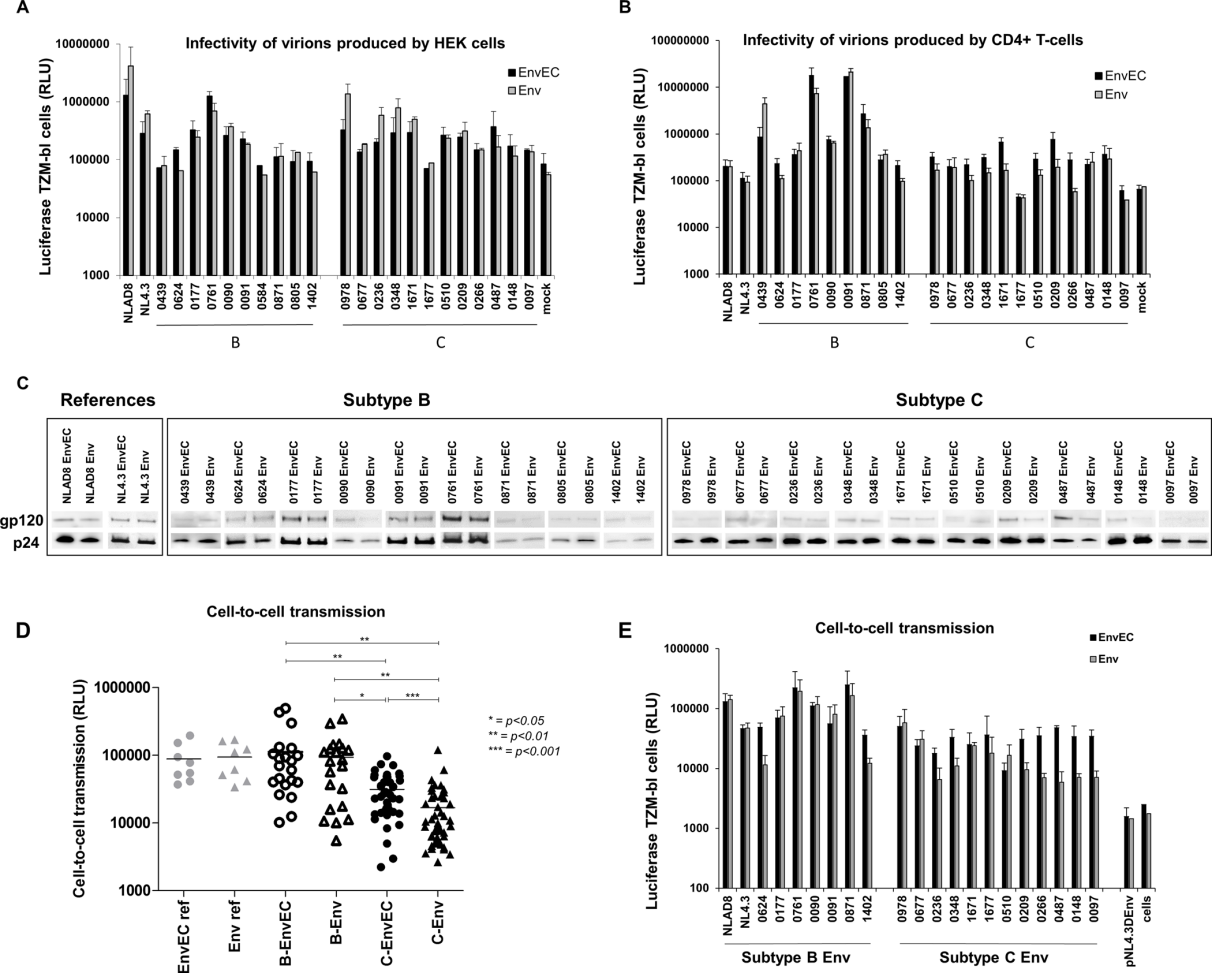
Infectivity of C-Env virions, Env incorporation in virions produced by CD4+ T-cells and cell-to-cell transmission are lessened. **(A and B) Infectivity of virions produced by HEK 293T cells (A) and CD4+ T-cells (B) in a single round TZM-bl assay.** TZM-bl cells (2x10^4^ cells/well) were infected with 2 ng/ml equivalent p24 of Env or EnvEC recombinant viruses produced from HEK 293T cells **(A)** or from CD4+ T-cells 5 days post infection **(B)**. Viral entry was monitored in cell lysates 48h post-infection. Infections were performed in triplicated wells. The means of three independent experiments with HEK293T cells and of 4 independent infections with CD4+ T cells from 4 different donors are shown. Error bars report standard deviation **C**. **Env incorporated in virions produced by CD4+ T-cells.** P24 and gp120 in day 5 supernatants of CD4+ T-cells were quantified by Western Blot. One representative experiment of two is shown. **D and E. Cell-to-cell transmission.** CD4+ T-cells were infected as above with 1 ng/ml equivalent p24. After 48 hours, cell were cocultured with TZM-bl cells in the presence of IDV to avoid any contribution of free virus. The next morning, MVC and AMD3100 were added to ensure single-round infection. Luciferase in TZM-bl cells was measured after 48 hours. **(D)** The mean and standard deviation of four independent experiments performed with CD4+ T-cells from four different donors are reported. **(E)** The same data is shown for each individual virus pair.

We then inquired whether the gp41CT affected the infectivity of C-Env virions compared to their C-EnvEC counterpart. We therefore measured the infectivity of Env and EnvEC recombinant pairs released by transfected HEK 293T cells and by infected CD4+ T-cells using a TZM-bl-based single-cycle assay. To this aim, TZM-bl cells were infected with equivalent amounts of Env and EnvEC recombinants (normalized to p24). As reported in Fig 5A, entry and the early steps of viral replication in TZM-bl cells of Env and EnvEC viruses produced in HEK 293T cells were comparable for most virus pairs, regardless of subtype and of the gp41CT (p>0.05 according to a paired t-test). Likewise, and in keeping with viral propagative infection, B-EnvEC and B-Env virions produced from CD4+ T-cells comparably infected TZM-bl cells in the single-round infection assay (Fig 5B). In contrast, entry of 8/12 C-Env viruses produced by CD4+ T cells was 2 to 5-fold lower than their C-EnvEC counterpart (p = 0.0097) (Fig 5B).

Given that the gp41CT mediates Env incorporation into assembling virions, we investigated whether differences in Env incorporation underly the lower infectivity of C-Env virions released by CD4+ T-cells. Unfortunately, no gp120 and very little or no p24 was detectable by Western Blot (WB) after sucrose gradient centrifugation of C-Env virus supernatants, at all time points. Therefore, unpurified viral supernatants were used to estimate Env incorporation by WB. B-Env, B-EnvEC and C-EnvEC viruses incorporated comparable Env levels while some C-Env viruses contained less Env (e.g. 0677, 1671, 0209) (Fig 5C). These results suggest that the gp41CT of subtype C affects viral entry, partly by less efficiently mediating incorporation of Env into virions produced in CD4+ T cells, akin to gp41ΔCT mutants [43, 44, 70, 90, 91]. However, Env in virions did not always correlate with entry of free virions into TZM-bl cells (e.g. strains 0677, 0487, 0148) (Fig 5B), suggesting that Env incorporation into virions is not the mechanism accounting for lower infectivity of virions or that some tolerance exists in terms of the number of Env spikes necessary to mediate Env.

### Cell-to-cell transmission of C-Env viruses is impaired

We next assessed whether the gp41CT and/or the lower levels of Env in virions affected cell-to-cell transmission. CD4+ T-cells infected with B- and C-EnvEC and Env recombinant virus pairs for 48 hours were co-cultured with TZM-bl cells in the presence of indinavir to avoid any concomitant infection by free virions. As reported in Fig 5D, transmission of virus pairs faithfully recapitulated replication capacity, with transmission of B-EnvEC = B-Env > C-EnvEC > C-Env viruses (p = 0.0002). Most interestingly, cell-to-cell transmission of C-Env viruses with poor replication capacity in CD4+ T-cells was considerably lower than that of the corresponding C-EnvEC recombinant while the difference was generally less for C-EnvEC/C-Env pairs with intermediate replication capacity (Fig 5E). Furthermore, viruses with low levels of Env (Fig 5C) generally displayed impaired cell-to-cell transmission, showing that decreased cell-to-cell transmission plays a fundamental role in the lower viral propagative infection of C-Env viruses. Infection of TZM-bl cells with the supernatant of the CD4+T-cell-TZM-bl co-culture yielded no signal (not shown), indicating that infection by free virus is negligible in this set-up.

### Subtype C MA decreases viral production, partially rescues viral replication in CD4+ T-cells but does not rescue infectivity

**Fig 6:**
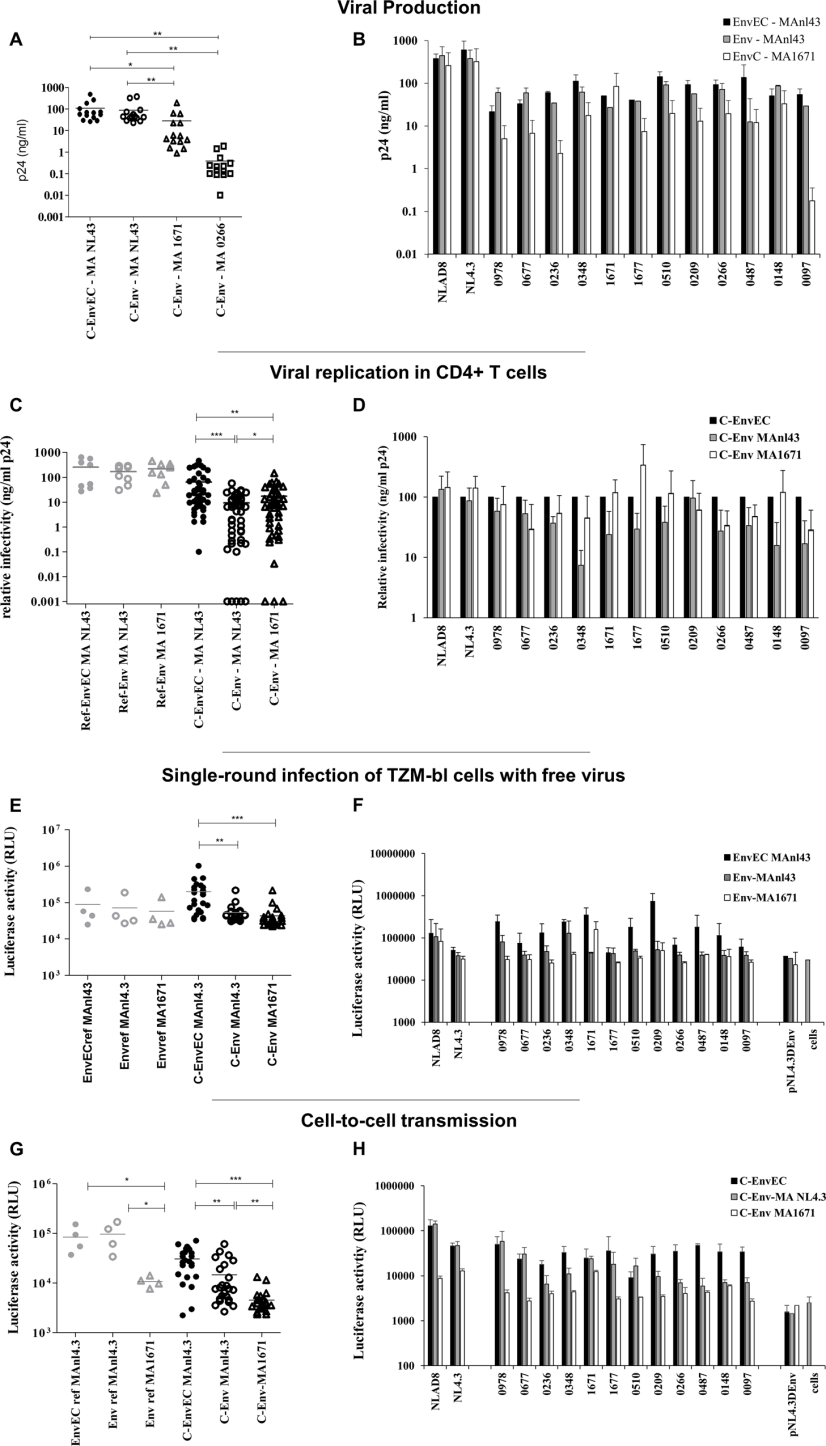
Subtype C MA decreases viral production, only partially rescues viral replication in CD4+ T-cells but does not restore infectivity. **A and B. Production of viruses containing subtype C MA.** The *matrix* sequences of two subtype C strains with intermediate replication capacity (MA_1671_) and with poor replication capacity (MA_0266_) were cloned into the EnvEC and Env backbones. Recombinant viruses with subtype C MAs were generated by transfecting HEK 293T cells with these backbones and the corresponding subtype C EnvEC and Env amplicons from patient samples. p24 produced was measured by ELISA 48 hours post transfection. At least three independent productions with MA_NL4.3_ and two with MA_1671_ and MA_0266_ are averaged and error bars show standard deviation. Results are shown for all viruses as groups (A) and per pairs (B). **C and D. Replication of C-EnvEC and C-Env recombinant viruses containing subtype C MA**_**1671**_ **in CD4+ T-cells.** CD4+ T-cells were infected with C-EnvEC and C-Env recombinants containing the MA of NL4.3 or of strain 1671, and p24 was measured in the supernatants 5 days post-infection. The average and standard deviation of four independent experiments performed with CD4+ T-cells from four different donors are shown. Results are shown for all viruses as groups (C) and per pairs (D) In (D), relative infectivity with respect to the corresponding C-EnvEC value is reported. **E and F. Infectivity of virions produced by CD4+ T-cells in a TZM-bl single round assay.** TZM-bl cells were infected with supernatants from infected CD4+ T-cells collected 5 days after infection and adjusted to equivalent p24 contents (2 ng/well). Infection was monitored 48h post-infection as in Fig 3. The mean of two independent experiments performed with CD4+ T-cell supernatants from two independent donors are shown and error bars report standard deviation. **G and H. Cell-to-cell transmission of viruses with subtype C MA**_**1671.**_ CD4+ T-cells were infected with C-EnvEC, C-Env or C-EnvMA_1671_ viral particles for 40 hours, extensively washed, then co-cultured with TZM-bl cells for a further 48 hours in the presence of IDV. MVC and AMD3100 were added the next morning. The mean of two independent experiments is shown. Error bars represent standard deviation. Panel **G** shows the same data as panel **F** detailed for each individual pair.

To assess whether the lower Env incorporation and infectivity of C-Env virions reflected a mismatch between subtype C gp41CT and subtype B MA, we cloned the MAs of two subtype strains into the pNL4.3ΔEnv backbone. We selected the MA sequences from one strain with intermediate replication capacity (strain 1671) and from one strain with poor replication capacity (strain 0266). With both subtype C MAs, C-Env viral production by transfected HEK 293T cells was lower than with MA_NL4.3_ (p = 0.0091 for MA_1671_ and p = 0.0106 for MA_0266_), despite the MA-gp41CT subtype match (Fig 6A and 6B).

Because of the low viral production with MA_0266_, CD4+ T-cells could not be infected with recombinants containing MA_0266_. In CD4+ T-cells infected with MA_1671_, MA_1671_ partially rescued viral replication in a donor-dependent fashion: for donor 1, MA_1671_ rescued replication fully for 2 Envs and partially for 4 Envs (from 4.3% to 81% of the corresponding C-EnvEC virus); for donor 2, replication was fully rescued for 5 Envs but not for the other 7; for donor 3, replication was rescued partially for 6/12 Envs (from 1.6% to 59% replication of the corresponding C-EnvEC virus) and for donor 4, infectivity was rescued for 3 strains and partially (4.9% and 29.4%) for two strains but not for the remaining 7 strains. Mean p24 in supernatants was 64.5 ng/ml for C-EnvEC viruses, 9.3 ng/ml for C-Env viruses with MA_NL4.3_ and 17.5 ng/ml for C-Env viruses with MA_1671_ (Fig 6C and 6D).

In single round infection assays using virions produced by CD4+ T-cells (normalized to p24), MA_1671_ partially rescued infectivity of the autologous C-Env_1671_ strain, (from 12.6% to 45.5%) but did not rescue infectivity of any other subtype C Env (Fig 6E and 6F). Env incorporation into virions with MA_1671_ could not be visualized by WB (not shown), in line with the low infectivity of these viruses. In cell-to-cell transmission assays, not only was MA_1671_ unable to rescue cell-to-cell transmission of all C-Envs viruses, but it further decreased cell-to-cell transmission compared to the C-Env virus (p = 0.0037, paired t-test), including for the autologous Env_1671_ and for the references strains NL4.3 and NLAD8 (Fig 6G and 6H).

Taken together, these data demonstrate that the MA-gp41CT subtype match alone cannot be held accountable for the low level of incorporation into virions of Envs with subtype C gp41CT. Rather, they confirm the driver role of MA in viral assembly and cell-to-cell transmission.

## Discussion

The results reported here show that viruses with subtype C gp41CTs have lower replicative capacity *in vitro* than viruses with the same Env extracellular domain but a subtype B gp41CT in CD4+ T-cells, indicating that polymorphisms in this region directly contribute to viral replication capacity. Lower replicative capacity was due to both lower infectivity of free viruses produced by CD4+ T-cells and for the main part, to lower cell-to-cell transmission (Fig 5), ultimately resulting in decreased viral propagation in CD4+ T-cell cultures (Fig 2A and 2B). Notably, decreased replication capacity *in vitro* was cell-type dependent (Fig 5A and 5B), as subtype C Envs lowered viral replicative capacity in CD4+ T-cells (Fig 2A–2D), but not in MDMs (Fig 2E–2G). This dichotomy is surprising, considering that gp41CT truncations inhibit viral replication in both CD4+ T-cells and macrophages [42–44, 70, 90, 91]. One possible explanation is that the slower assembly process in this cell type, the lesser dependence of transmission on Env [107] and the retention of virions in PM-related invaginations or in Virus Containing Compartments [108–112] may compensate for poor virion infectivity in MDMs. Of note, an earlier report had described lower replication of subtype C primary strains in MDMs, with a peak at day 21, which never reached the levels of p24 secreted by subtype B strains [113]. Although the replication kinetics we record for C-Env viruses in CD4+ T-cells recapitulated those reported by Constantino in MDMs [113], we did not record any difference in replication kinetics between B-Env and C-Env viruses in infected MDMs (Fig 2E–2G). In line with this observation, donor-dependent viability was also minimal (not shown). Given that the recombinant viruses used in our study are based on a subtype B backbone and differ only by the gp41CT, these different results suggest that viral proteins other than Env account for the difference in replication capacity between subtypes.

Lower viral infectivity of C-Env viruses in CD4+ T-cells could be associated with lower Env levels in virions for some strains, particularly for some of the viruses displaying the lowest replicative capacities, such as 0209 and 0266 (Fig 5C). This defect could not be attributed to altered intracellular localization of Env (not shown). Furthermore, such an observation did not hold true for all C-Env virions. Alternatively, it is also possible that less favorable Env conformations governed by the gp41CT [69–73] contribute to infectivity and cell-to-cell transmission. Further structural studies would be warranted to assess whether Env conformation and/or the number of Env spikes at the surface of virions varies across subtypes, as such a finding would have implications for vaccine design. Given that cell-to-cell transmission requires the production of virus particles with appropriate Env levels [8], it is not surprising that both infection of free virions and cell-to-cell transmission are affected by the low levels of Env incorporated into virions, nor that lower Env levels in virions preferentially affect cell-to-cell transmission. Cell-to-cell transmission is the predominant mode of HIV-1 spread in CD4+ T lymphocytes and is thougt to be 100–1000 times more efficient than infection by cell-free virus [5, 10, 114–118]. Our results showing that cell-to-cell transmission best recapitulates viral replication in CD4+ T-cells is in line with this observation and with Env:p24 levels. *In vivo*, cell-to-cell transmission is belived to play a crucial role due to the tight packing of CD4+ T-cells in lymph nodes [119]. Because TZM-bl cells express high levels of CD4, the differences reported here might be underestimated, and it is likely that they might have a much stronger impact *in vivo*.

A recent study based on sequencial gp41CT truncations dissociated the determinants crucial for cell-free infection from those permitting cell-to-cell transmission of virus, showing that large gp41CT truncations (>92 AA) affect cell-to-cell transmission while short gp41CT truncations (<43 AA) are detrimental to cell-free infection [120]. This study also provides evidence that mutations at the Y_802_W_803_ diaromatic motif or at the LL_800_ dileucine motifs involved in TIP47 and prohibitin binding respectively [54, 121] affect cell-free virus infection but not cell-to-cell propagation [120]. In contrast to this report, we found that both cell-free and cell-to-cell fusion of viruses with subtype C gp41CT were affected, although to different degrees for different viral strains (e.g. strains 1671, 0510, 0487, 0148, 0097), and that cell-to-cell transmission did not compensate for poor free-virion infectivity. One possible explanation for these contrasting results could reflect the fact that none of the subtype C variants included in our study displayed gp41CT truncations nor the LL_800_RQ mutation engineered by Durham [120]. All subtype C gp41CT sequences associated with lower viral replication harbored a YW_796_YL polymorphism accompanied by L_800_F polymorphism (S1 Fig) at the compensatory position described by Qi et al to restore the interaction of the gp41CT with FIP1C/Rab11a and Env packaging [55, 66]. Although the polymorphisms observed in subtype C patient sequences differ from the mutations introduced by Qi et al [66], their role in Env packaging and virus replication would warrant further investigation.

Replication of C-Env viruses in CD4+ T cells was only very partially rescued by subtype C MA (Fig 6C and 6D). While MA_1671_ rescued replication of a virus harboring the autologous Env_1671_, it only very partially and not consistently rescued replication of other C-Env viruses (Fig 6C and 6D). Furthermore, the subtype match between MA and the gp41CT failed to rescue infectivity in single-round infections (Fig 6E and 6F) and, surprisingly, further impaired cell-to-cell transmission of viruses with subtype C Env. These findings differ from a previous case study documenting the co-evolution between MA and the gp41CT optimizing Env incorporation [91]. We also found that subtype C MA further decreased (MA_1671_) or nearly abrogated (MA_0266_) viral production (Fig 6A and 6B), in sharp contrast with Beaumont’s report [91]. In the case reported by Beaumont, poor Env incorporation was consequent to a truncation of LLP-1 [91], while in our study none of the gp41CT patient sequences harbored truncations in LLP-1 or in LLP-2/3 (S1 Fig), which may limit the rescue potential by MA. Patients 0266 and 1671 had comparable viral loads and CD4 counts, had been infected for a comparable number of years, and displayed no particular mutation in the MA sequence (S2 Fig), which could explain such drastic differences. Our findings therefore imply that the lower infectivity of C-Env virions is not due to the subtype match with MA but rather reflects intrinsic properties of the gp41CT and that other mechanisms must operate. Further studies will be needed to identify them. They also further underscore that the viral MA is the main driver of viral assembly and replication capacity.

Intracellular viral protein contents 5-days post infection were low and mature forms of Gag were absent in cultures infected with C-Env viruses (Fig 3A). While lower viral protein contents did not correlate with β-actin expression, arguing against cell-death as a cause of lower viral production, nor with impaired viral release (Fig 4), it most likely reflects the low replication capacity and lack of spread of C-Env viruses in CD4+ T-cells. It is also unlikely that low viral protein contents in cell lysates reflect lower protein expression, given that at early time points (40 hours post infection) intracellular viral protein levels were comparable (Fig 3B). This conclusion is reinforced by the observation that Envs with subtype B or subtype C gp41CTs do not trigger any transcription from the LTR nor activate NF-κB (Beraud and Lemaire, submitted), in contrast to previous reports by other groups [83–86].

In our experimental design, recombination between the Env or EnvEC backbone and the Env or EnvEC amplicon occurs within Vpu, although it is not known where the breakpoint occurs. Vpu is a frequent breakpoint for inter-subtype recombination *in vivo* [122] and may impact its efficiency in down modulating BST-2 [122, 123] and viruses with recombinant BC [123] and BF [122] Vpu are moderately more efficient than subtype B or CB recombinant Vpu in down-modulating BST-2. In our study, viral release tended to be higher for viruses with subtype C gp41CT than for their isogenic counterpart with the gp41CT of NL4.3 (Fig 4). Moreover, 5 days post infection, mature Gag products were lower in CD4+ T-cell lysates infected with C-Env viruses (Fig 3), which is inconsistent with a defect in viral release [124].

Polymorphisms in the gp41CT also overlap the second exon of Tat and Rev. With the exception of one subtype B Tat, which displayed the same STOP codon as HXB2, no premature STOP codon was present. Tat was highly conserved, particularly the basic AA, except for a K13E mutation in the second exon in many, but not all, subtype C Tat sequences (S3A Fig). A defect in Tat function would have impacted luciferase induction in TZM-bl cells, and is thus not consistent with the comparable signal induced by HEK293T supernatants. Rev sequence inspection highlighted that subtype C sequences were 8 AA shorter than subtype B, and two (those which did not contain the 7 AA insertion) were 15 AA shorter. Amino acids involved in Rev multimerization (Ile52, Ile55 and Ile59) as well as the Arginine-rich RNA-binding effector domain were highly conserved. The Leucine rich effector domain displayed two L⇾I mutations, and was not affected by the 7 AA insertion. The Nuclear Export Signal was highly conserved and was not affected by the 7 AA insertion, which lies downstream (S3B Fig). Furthermore, a Rev-related defect in Env mRNA export is not consistent with the higher subtype C Env expression levels in infected CD4+ T-cells (Fig 3) nor with the similar viral production by HEK293T cells recorded for all recombinants (not shown). Last but not least, in macrophages, where Rev plays a crucial role, no difference in replicaton was recorded, suggesting that the observed impact reflects a gp41CT-mediated effect rather than a Tat-related transcriptional defect or Rev-dependent impaired viral protein export.

Why subtype C strains would have evolved to reach lower replication levels, and how polymorphisms in this region would benefit the virus remains to be established. One possibility is that polymorphisms in this region reduce the immunogenic or cytopathic potential of Env, and therefore represent an advantage in terms of immune escape thereby favoring the long term persistence of infection. In macaques infected with SIVmac bearing a Y_721_xxL mutation that increased Env expression, viral load was controlled by the immune response until reversion of the mutation [125]. Env is cytopathic, pro-apoptotic and is targeted by the humoral immune response [126–128]. In our experiments, CD4+ T cell death was related to the level of infectivity rather than to a specific Env or Env gp41CT (data not shown), supporting this hypothesis. Higher Env tolerance, delayed replication kinetics, decreased Env in virions and immune escape could explain why subtype C strains account for over half of worldwide infections.

In conclusion, our results show that polymorphisms in the gp41CT of subtype C decrease viral infectivity and cell-to-cell transmission, thereby impairing viral propagative infection in CD4+ T-cells, most likely by enabling less Env incorporation into virions. Subtype C MA only partially rescued viral replication capacity, in a strain-dependent manner, emphasizing its crucial role as a driver of viral assembly. One compensatory mechanism evolved by subtype C strains could involve higher Env levels and tolerance, favoring the long term persistence of infection, particularly via macrophages. Other compensatory mechanisms may exist elsewhere in the subtype C viral genome, such as intrinsic properties of MA or promoter-related replication advantages [129, 130] linked to AP-1 and the third NF-κB site typically found in the LTR of subtype C strains [130–132]. These findings may have consequences for vaccine design as they imply subtype-dependent differences.

## Supporting Information

S1 Fig Alignment of subtype B and subtype C gp41CT sequences against the NL4.3 reference. The patient gp41CT were sequenced from the same *env* amplicons used to produce Env-recombinant viruses to avoid PCR-selection biases. Sequencing was performed using BigDye Terminator v3.1 dye on a Applied Biosystems 3500 xL DX genetic analyzer (Applied Biosystems Europe BV, Belgium), with sense primer AV326 and reverse primer AV331 [104]. Sequence alignments were performed by CLC Main Workbench v7.5 Software (Aarhus, Denmark). The domains involved in Env trafficking (Y_712_SPL, C-terminal LL_856_ and Y_802_W_803_,) are boxed (yellow and green) and topped with a star, the P_T/R_RIR sequence (blue) and the three immunogenic epitopes are boxed (pink). The locations of the LLP α-helices are assigned based on the NL4.3 reference sequence. gp41CT sequence analysis highlights that subtype B strains closely resembles the NL4.3 reference, whereas subtype C harbors a number of specific polymorphisms. The main Y_712_SPL endocytic motif (yellow box), the Y_802_W_803_ diaromatic motif (green box) as well as all but one Arg spanning the LLP α-helices, the Arg-rich P_T/R_RIR motif (blue box) and Cys residues within LLP-1 are highly conserved in all samples, underscoring their chief role in Env intracellular traffic and incorporation into virions. The second Y_768_XXL motif is 100% conserved as well. Notably, the C-terminal dileucine motif LL_856_ within LLP-1 (yellow box) is replaced by LQ_856_ in 9/12 subtype C Envs (8 pure, and 1 LL/LQ_856_ mixtures). Other subtype C-specific polymorphisms involve the dileucine motifs spanning the gp41CT LLP-2/3 α-helices (LLL_776_→FIL_776_ and LL_800_→LV_800_), polar/charged residues (WN_798_→GS_798_, SQ_805_→GL_805_, N_809_→K, NA_817_→DT_817_ and R_853_→A in LLP-1) and a conserved seven AA insertion (SSLRGLQ, 2 α-helical turns) between R_787_ and R_788_ (10/12 subtype C Envs). The Kennedy sequence contains a number of subtype-specific mutations, including a R→Q and D→N/S/G mutations in the E_739_RDRD_743_ epitope.
(TIF)

S2 Fig Sequence alignment of subtype C strain MA against the NL4.3 reference. MA was sequenced from the same RNA extracted and used for Env amplification. A cDNA was synthesized from 10 μl RNA in a one-step PCR reaction using forward primer KVL064 and reverse primer KVL079 [133] as described in [133]. Two microliters of cDNA were further amplified using Forward primer KVL066 and Reverse primer KVL080 [133]. Amplicon size and quality was verified by agarose gel electrophoresis and sequenced using primers KVL066, KVL080, KVL081 and GA1 [133]. Sequences were aligned and analyzed using the CLC Bio Main Workbench 6.82 software. The consensus sequence logos were generated with WebLogo3.3. All residues known to be involved in the interaction of MA with Env and in Env incorporation into virions (i.e. residues L_8_ [8, 81], L_12_, L_30_, V_34_ [37, 43], K_32_ [41], L_49_ [134], E_99_ [135], the basic domain of MA (AA 17–21) [103]) were 100% conserved in all subtype C strains, with the exception of S_8_ [8, 81], which was replaced by an Arg in all subtype C sequences, and of residue L_30_, which was conserved in 8/12 of strains and was replaced by a Met in the remaining 4 viruses, but could not be associated with lower replication levels or Env incorporation. MA compensatory mutations V34I [37, 43, 91] and Q62R [136] were consistently absent from subtype C MAs. S9R was present in 11/12 subtype C strains and S9K in one, regardless of replication capacity, and the role of this specific polymorphism without a mutation at L_8_ is not known. Basic residues 17–21 mediating MA interaction with Env [137] [38, 40–42, 44, 70, 138] or AA involved in p55Gag trafficking via adaptor proteins (Y_132_ and V_135_ at the MA/CA junction) [49, 68, 139, 140] were also conserved. AA involved in myristylation (AA1-6 and G_10_), in the myristyl switch (H_89_) or in p55Gag targeting to the PM (AA 84–89) [141–146] were conserved, and E12 hosted a Lysine, as reported for HIV-2 [146]. Other subtype C specific polymorphisms were generally found in all sequences and we could not identify polymorphisms that were only present in strains with very poor replication capacity or that were associated with the presence of subtype C polymorphisms within the gp41CT.
(TIF)

S3 Fig Sequence alignment of subtype B and C Tat and Rev sequences against the NL4.3 reference. **Tat (A) and Rev (B) exon II sequence alignments.** The second exon of Tat and of Rev overlap the gp41CT. Tat and Rev sequences were aligned against the NL4.3 reference using the CLC Bio Main Workbench v.7.5 software. Tat was highly conserved, particularly the basic AA, with the exception of a K13 E mutation in the second exon in many, but not all, subtype C Tat sequences. Rev subtype C sequences had a CAA (Gln) TAA (STOP) mutation matching the HXB2 premature end (marked _with_ a *). Therefore, subtype C Rev proteins were 8 AA shorter than subtype B, and two (those which did not contain the 7 AA insertion) were 15 AA shorter. Amino acids involved in Rev multimerization (I_52_, I55 and I_59_) and the Arginine-rich RNA-binding effector domain were generally conserved. The Nuclear Export Signal displayed two LI mutations and was not affected by the 7 AA insertion.
(TIF)
