# Supplementary figures and images for: The Envelope Cytoplasmic Tail of HIV-1 Subtype C Contributes to Poor Replication Capacity through Low Viral Infectivity and Cell-to-Cell Transmission
Source: PLoS One. 2016 Sep 6;11(9):e0161596. doi: 10.1371/journal.pone.0161596 (PMC5012655; doi:10.1371/journal.pone.0161596)

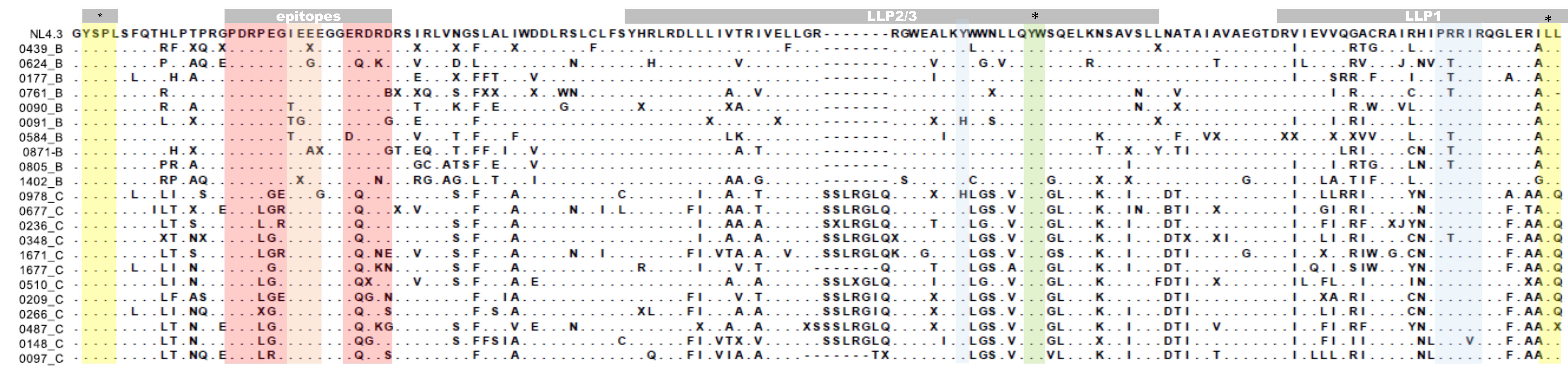

Supplement: S1 Fig — The patient gp41CT were sequenced from the same env amplicons used to produce Env-recombinant viruses to avoid PCR-selection biases. Sequencing was performed using BigDye Terminator v3.1 dye on a Applied Biosystems 3500 xL DX genetic analyzer (Applied Biosystems Europe BV, Belgium), with sense primer AV326 and reverse primer AV331 [104]. Sequence alignments were performed by CLC Main Workbench v7.5 Software (Aarhus, Denmark). The domains involved in Env trafficking (Y712SPL, C-terminal LL856 and Y802W803,) are boxed (yellow and green) and topped with a star, the PT/RRIR sequence (blue) and the three immunogenic epitopes are boxed (pink). The locations of the LLP α-helices are assigned based on the NL4.3 reference sequence. gp41CT sequence analysis highlights that subtype B strains closely resembles the NL4.3 reference, whereas subtype C harbors a number of specific polymorphisms. The main Y712SPL endocytic motif (yellow box), the Y802W803 diaromatic motif (green box) as well as all but one Arg spanning the LLP α-helices, the Arg-rich PT/RRIR motif (blue box) and Cys residues within LLP-1 are highly conserved in all samples, underscoring their chief role in Env intracellular traffic and incorporation into virions. The second Y768XXL motif is 100% conserved as well. Notably, the C-terminal dileucine motif LL856 within LLP-1 (yellow box) is replaced by LQ856 in 9/12 subtype C Envs (8 pure, and 1 LL/LQ856 mixtures). Other subtype C-specific polymorphisms involve the dileucine motifs spanning the gp41CT LLP-2/3 α-helices (LLL776→FIL776 and LL800→LV800), polar/charged residues (WN798→GS798, SQ805→GL805, N809→K, NA817→DT817 and R853→A in LLP-1) and a conserved seven AA insertion (SSLRGLQ, 2 α-helical turns) between R787 and R788 (10/12 subtype C Envs). The Kennedy sequence contains a number of subtype-specific mutations, including a R→Q and D→N/S/G mutations in the E739RDRD743 epitope. (TIF) [file pone.0161596.s001.tif]

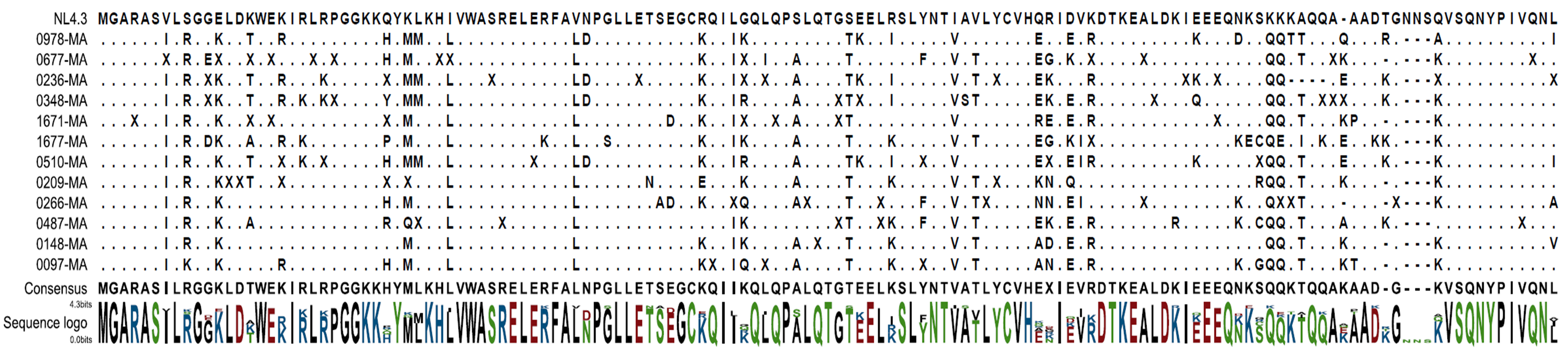

Supplement: S2 Fig — MA was sequenced from the same RNA extracted and used for Env amplification. A cDNA was synthesized from 10 μl RNA in a one-step PCR reaction using forward primer KVL064 and reverse primer KVL079 [133] as described in [133]. Two microliters of cDNA were further amplified using Forward primer KVL066 and Reverse primer KVL080 [133]. Amplicon size and quality was verified by agarose gel electrophoresis and sequenced using primers KVL066, KVL080, KVL081 and GA1 [133]. Sequences were aligned and analyzed using the CLC Bio Main Workbench 6.82 software. The consensus sequence logos were generated with WebLogo3.3. All residues known to be involved in the interaction of MA with Env and in Env incorporation into virions (i.e. residues L8 [8, 81], L12, L30, V34 [37, 43], K32 [41], L49 [134], E99 [135], the basic domain of MA (AA 17–21) [103]) were 100% conserved in all subtype C strains, with the exception of S8 [8, 81], which was replaced by an Arg in all subtype C sequences, and of residue L30, which was conserved in 8/12 of strains and was replaced by a Met in the remaining 4 viruses, but could not be associated with lower replication levels or Env incorporation. MA compensatory mutations V34I [37, 43, 91] and Q62R [136] were consistently absent from subtype C MAs. S9R was present in 11/12 subtype C strains and S9K in one, regardless of replication capacity, and the role of this specific polymorphism without a mutation at L8 is not known. Basic residues 17–21 mediating MA interaction with Env [137] [38, 40–42, 44, 70, 138] or AA involved in p55Gag trafficking via adaptor proteins (Y132 and V135 at the MA/CA junction) [49, 68, 139, 140] were also conserved. AA involved in myristylation (AA1-6 and G10), in the myristyl switch (H89) or in p55Gag targeting to the PM (AA 84–89) [141–146] were conserved, and E12 hosted a Lysine, as reported for HIV-2 [146]. Other subtype C specific polymorphisms were generally found in all sequences and we could not identify polymorphisms that we [file pone.0161596.s002.tif]

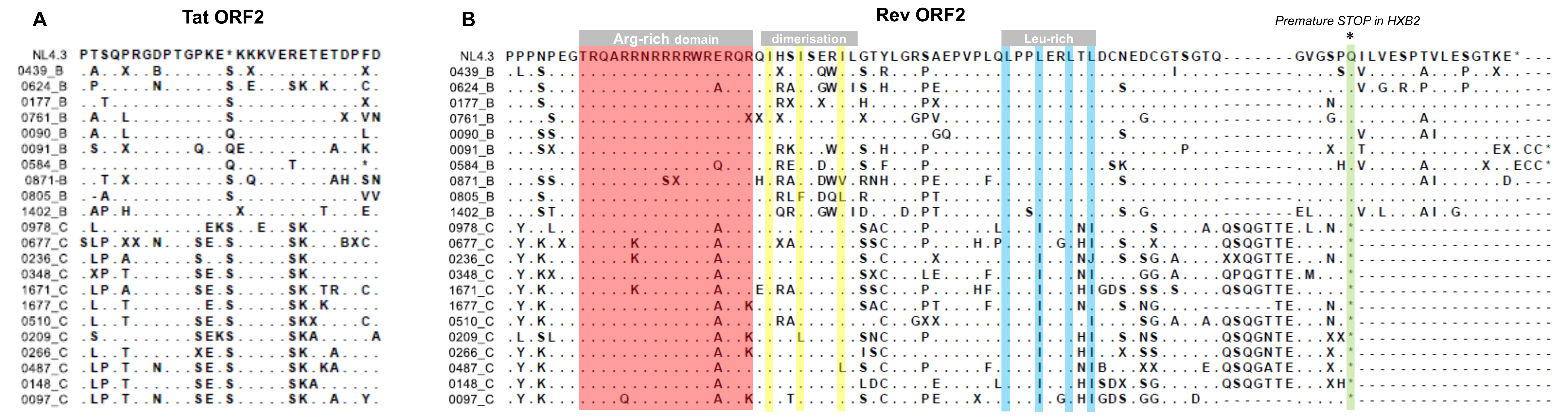

Supplement: S3 Fig — Tat (A) and Rev (B) exon II sequence alignments. The second exon of Tat and of Rev overlap the gp41CT. Tat and Rev sequences were aligned against the NL4.3 reference using the CLC Bio Main Workbench v.7.5 software. Tat was highly conserved, particularly the basic AA, with the exception of a K13 E mutation in the second exon in many, but not all, subtype C Tat sequences. Rev subtype C sequences had a CAA (Gln) TAA (STOP) mutation matching the HXB2 premature end (marked with a *). Therefore, subtype C Rev proteins were 8 AA shorter than subtype B, and two (those which did not contain the 7 AA insertion) were 15 AA shorter. Amino acids involved in Rev multimerization (I52, I55 and I59) and the Arginine-rich RNA-binding effector domain were generally conserved. The Nuclear Export Signal displayed two LI mutations and was not affected by the 7 AA insertion. (TIF) [file pone.0161596.s003.tif]
